# **EZH2-mediated epigenetic suppression of long noncoding RNA SPRY4-IT1 promotes NSCLC cell proliferation and metastasis by affecting the epithelial–mesenchymal transition**

**DOI:** 10.1038/cddis.2014.256

**Published:** 2014-06-26

**Authors:** M Sun, X-H Liu, K-H Lu, F-Q Nie, R Xia, R Kong, J-S Yang, T-P Xu, Y-W Liu, Y-F Zou, B-B Lu, R Yin, E-B Zhang, L Xu, W De, Z-X Wang

**Affiliations:** 1 Department of Biochemistry and Molecular Biology, Nanjing Medical University, Nanjing, People's Republic of China; 2 Department of Oncology, First Affiliated Hospital, Nanjing Medical University, Nanjing, People's Republic of China; 3 Department of Oncology, Nanjing First Hospital, Nanjing Medical University, Nanjing, People's Republic of China; 4 Department of Obstetrics and Gynecology, First Affiliated Hospital, Nanjing Medical University, Nanjing, People's Republic of China; 5 Department of Oncology, Second Affiliated Hospital, Nanjing Medical University, Nanjing, People's Republic of China; 6 Department of Thoracic Surgery, Nanjing Medical University Affiliated Cancer Hospital, Cancer Institute of Jiangsu Province, Nanjing, People's Republic of China

## Abstract

Recent evidence indicates that long noncoding RNAs (lncRNAs) have a critical role in the regulation of cellular processes such as differentiation, proliferation, and metastasis. These lncRNAs are dysregulated in a variety of cancers and many function as tumor suppressors; however, the regulatory factors involved in silencing lncRNA transcription are poorly understood. In this study, we showed that epigenetic silencing of lncRNA SPRY4 intronic transcript 1 (*SPRY4-IT1*) occurs in non-small-cell lung cancer (NSCLC) cells through direct transcriptional repression mediated by the Polycomb group protein enhancer of zeste homolog 2 (EZH2). *SPRY4-IT1* is derived from an intron within *SPRY4*, and is upregulated in melanoma cells; knockdown of its expression leads to cell growth arrest, invasion inhibition, and elevated rates of apoptosis. Upon depletion of EZH2 by RNA interference, *SPRY4-IT1* expression was restored, and transfection of *SPRY4-IT1* into NSCLC cells resulted in a significant antitumoral effect, both in culture and in xenografted nude mice. Moreover, overexpression of *SPRY4-IT1* was found to have a key role in the epithelial–mesenchymal transition through the regulation of E-cadherin and vimentin expression. In EZH2-knockdown cells, which characteristically showed impaired cell proliferation and metastasis, the induction of *SPRY4-IT1* depletion partially rescued the oncogenic phenotype, suggesting that *SPRY4-IT1* repression has an important role in EZH2 oncogenesis. Of most relevance, translation of these findings into human NSCLC tissue samples demonstrated that patients with low levels of *SPRY4-IT1* expression had a shorter overall survival time, suggesting that *SPRY4-IT1* could be a biomarker for poor prognosis of NSCLC.

Non-small-cell lung cancer (NSCLC) is the predominant form of lung cancer and accounts for the majority of cancer deaths worldwide, which includes adenocarcinomas and squamous cell carcinomas.^1^ Despite recent advances in clinical and experimental oncology, the prognosis of NSCLC remains poor, with a 5-year overall survival rate of around 11%.^2^ A continuing problem of NSCLC tumorigenesis is the invasion and metastasis of cancer cells, which is the main cause of death in patients. Therefore, a detailed understanding of the mechanisms and molecular pathways activated in metastatic cells is crucial in identifying new treatment options for anticancer therapy that target metastasis.

During the past decade, large-scale sequencing efforts and the ENCODE project have revealed that a large fraction of the human noncoding genome is transcribed.^3^ Despite primary transcripts covering 75% of the human genome, most of these are noncoding transcripts that yield long noncoding RNAs (lncRNAs), and only around 2% of the genome encodes proteins.^4, 5^ Recent extensive annotation of lncRNAs has been performed in multiple model organisms, revealing that they are often expressed in a spatial- and temporal-specific pattern.^6^ Although very few lncRNAs have been characterized in detail, they are known to participate in a wide range of biological processes, including the modulation of apoptosis and invasion, the reprogramming of stem cell pluripotency, and parental imprinting.^7, 8, 9^ In addition, lncRNA dysregulation has been linked to a diverse range of human diseases, in particular cancers. Although the expression levels of lncRNAs, such as maternally expressed gene 3, *GAS5*, and lncRNA-LET, were very low in some cancers, this was sufficient to repress tumor suppressor function.^10, 11, 12^

Similar to the regulation of mRNA transcription, some key transcription factors were shown to be involved in regulating lncRNA transcription; for example, p53 promotes lncRNA-p21 transcription and induces cell growth arrest and apoptosis, while E2F1 regulates lncRNA ERIC expression and influences the cellular response to DNA damage.^13, 14^ In addition, epigenetic modification has been found to play an important role in repressing lncRNA transcription. Hypermethylation of the promoter or intergenic differentially methylated region was shown to contribute to the inactivation of lncRNA maternally expressed gene 3 transcription in multiple tumors.^15^ Moreover, hypoxia-induced histone deacetylase 3 represses lncRNA-LET by reducing the histone acetylation-mediated regulation of its promoter region.^12^ Enhancer of zeste homolog 2 (EZH2), another important epigenetic regulatory factor, mediates modifications in histone methylation resulting in the repression of numerous tumor suppressor genes, such as *DAB2IP*, *CDH1*, and *DKKI*, as well as tumor suppressor microRNAs including miR-101, let-7c, and miR-200b.^16, 17^ However, it is not clear whether EZH2 also mediates the activation of low lncRNA transcription through lysine 27 residue of histone H3 trimethylation (H3K27me3) modification.

EZH2 is a methyltransferase and the core catalytic subunit of polycomb repressive complex 2. It has an essential role in the epigenetic maintenance of the H3K27me3 repressive chromatin mark.^18^ Overexpression of EZH2 has been widely reported in many cancers, including NSCLC.^16, 19, 20^ In the present study, we demonstrated that the lncRNA SPRY4 intronic transcript 1 (*SPRY4-IT1*), derived from an intron within *SPRY4*, is transcriptionally repressed by EZH2. *SPRY4-IT1* was previously reported to be upregulated in melanoma cells, and knockdown of its expression led to cell growth arrest, invasion inhibition, and elevated rates of apoptosis.^21^ Moreover, aberrant expression of *SPRY4-IT1* was also found to contribute to the abnormal condition of trophoblast cells HTR-8/Svneo.^22^ In this study, we explored the expression pattern of *SPRY4-IT1* in NSCLC tissues and cell lines, and investigated the effects of *SPRY4-IT1* expression on NSCLC cell phenotypes both *in vitro* and *in vivo*. Furthermore, we also showed that alteration of *SPRY4-IT1* expression can influence the expression levels of E-cadherin and vimentin proteins, indicating that *SPRY4-IT1* affects NSCLC cell proliferation and metastasis partly via the epithelial–mesenchymal transition (EMT). This study advances our understanding of the role of lncRNAs such as *SPRY4-IT1* as regulators of NSCLC pathogenesis, and facilitates the development of lncRNA-directed diagnostics and therapeutics.

## Results

### *SPRY4-IT1* expression was downregulated and correlated with a poor prognosis of NSCLC

**Figure 1:**
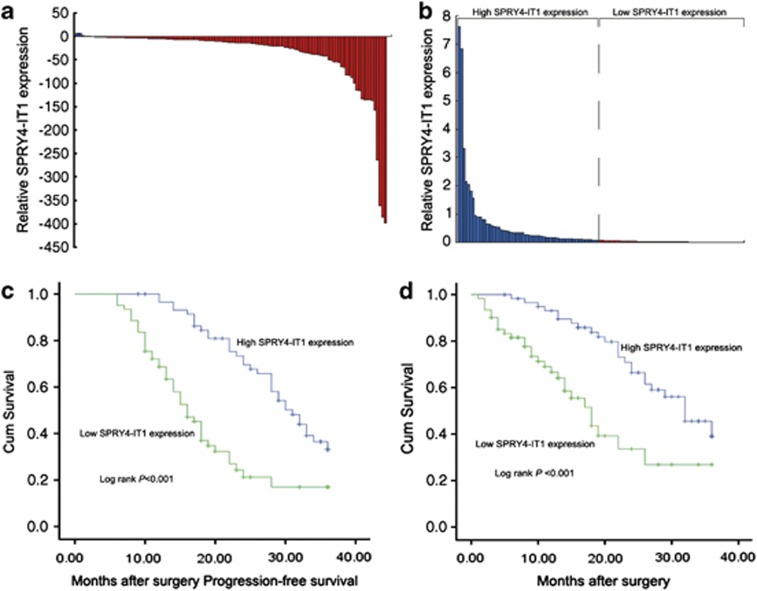
Relative SPRY4-IT1 expression in NSCLC tissues and its clinical significance. (**a**) Relative expression of *SPRY4-IT1* in NSCLC tissues (*n*=121) compared with corresponding nontumor tissues (*n*=121). *SPRY4-IT1* expression was examined by qPCR and normalized to *GAPDH* expression. Results are presented as the fold change in tumor tissues relative to normal tissues. (**b**) *SPRY4-IT1* expression was classified into two groups. (**c** and **d**) Kaplan–Meier disease-free survival and overall survival curves according to *SPRY4-IT1* expression levels. *P<0.05, **P<0.01

*SPRY4-IT1* expression levels were investigated in 121 paired NSCLC samples and adjacent histologically normal tissues using the quantitative PCR (qPCR). *SPRY4-IT1* expression was significantly downregulated (*P*<0.01) in 94.2% (114/121) of cancerous tissues compared with normal tissues (Figure 1a). *SPRY4-IT1* expression levels in NSCLC significantly correlated with tumor size (*P*=0.001), advanced pathological stage (*P*<0.001), and lymph node metastasis (*P*=0.003), but were not associated with other parameters such as gender (*P*=0.298) or age (*P*=0.522) in NSCLC (Supplementary Table 1).

### Association between *SPRY4-IT1* expression and patient survival

Kaplan–Meier survival analysis was conducted to investigate the correlation between *SPRY4-IT1* expression and NSCLC patient prognosis. According to relative *SPRY4-IT1* expression in tumor tissues, the 121 NSCLC patients were classified into two groups: the high *SPRY4-IT1* group (*n*=60, fold change ≤ median) and the low *SPRY4-IT1* group (*n*=61, fold change ≥ median) (Figure 1b). Progression-free survival was 33.1% for the high *SPRY4-IT1* group and 17% for the low *SPRY4-IT1* group, with median survival times of 31 and 16 months, respectively (Figure 1c). The overall survival rate over 3 years for the high *SPRY4-IT1* group was 39.1% *versus* 26.9% for the low *SPRY4-IT1* group, with median survival times of 32 and 18 months, respectively (Figure 1d).

Univariate analysis identified lymph node metastasis, tumor size, and *SPRY4-IT1* expression level as prognostic factors. Other clinicopathological features such as gender and age were not significant (Supplementary Table 2). Multivariate analysis of the three prognosis factors confirmed that a low *SPRY4-IT1* expression level was an independent predictor of poor survival for NSCLC (*P*=0.009), in addition to tumor node metastasis stage (*P*=0.041) (Supplementary Table 3).

### EZH2 repressed *SPRY4-IT1* expression via H3K27me3 modification

**Figure 2:**
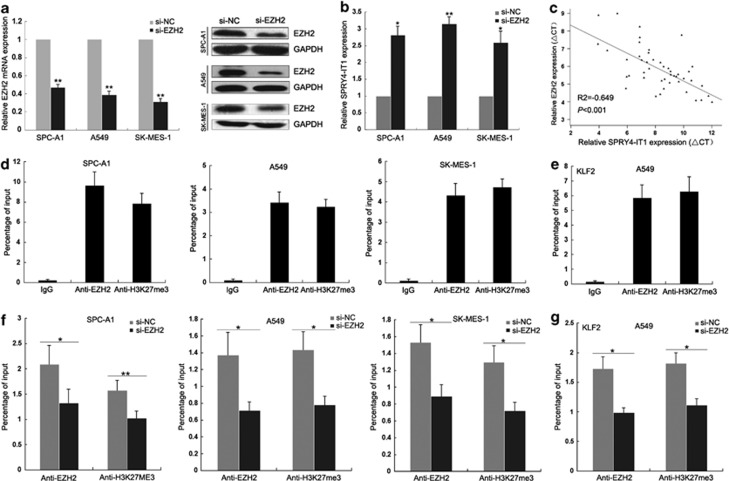
EZH2 is involved in *SPRY4-IT1* downregulation. (**a**) qPCR and western blot analysis of EZH2 expression levels following SPC-A1, A549, and SK-MES-1 cell treatment with si-EZH2. (**b**) qPCR analysis of *SPRY4-IT1* expression levels following SPC-A1, A549, and SK-MES-1 cell treatment with si-EZH2. (**c**) Analysis of the relationship between *SPRY4-IT1* and EZH2. (**d** and **e**) Chromatin immunoprecipitation (ChIP)–qPCR of EZH2 occupancy and H3K27-3me binding in the *SPRY4-IT1* promoter in three NSCLC cell lines. *KLF2* (a known EZH2 target gene) was used as a positive control and IgG as a negative control. (**f** and **g**) ChIP–qPCR of EZH2 occupancy and H3K27-3me binding in the *SPRY4-IT1* promoter in three NSCLC cell lines treated with EZH2 small interfering RNA (48 h) or scrambled small interfering RNA. *P<0.05, **P<0.01

Compared with 16HBE cells, qPCR showed that relative expression levels of *SPRY4-IT1* were reduced in NSCLC cells (Supplementary Figure S1a). Because of the different expression patterns for *SPRY4-IT1* in NSCLC and melanomas, we next investigated the mechanisms controlling tissue-specific expression of *SPRY4-IT1* by analyzing its promoter region. We identified one CpG island, and 5-Aza-2′-deoxycytidine (5-Aza) treatment of NSCLC cells significantly upregulated *SPRY4-IT1* expression (Supplementary Figure S1b). However, bisulfite sequencing revealed no difference between 5-Aza-treated NSCLC cells and control cells (Supplementary Figure S1c). Furthermore, knockdown of EZH2 to investigate whether histone methylation modification contributes to the decrease in *SPRY4-IT1* expression showed that *SPRY4-IT1* expression was instead promoted in NSCLC cells (Figures 2a and b and Supplementary Figure S2c).

The expression level of EZH2 was negatively correlated with *SPRY4-IT1* expression levels in NSCLC tissues (Figure 2c), and increased EZH2 expression in NSCLC tissues significantly correlated with tumor size, advanced pathological stage, and lymph node metastasis (Supplementary Table 5). Moreover, to investigate whether EZH2 could directly bind in the promoter region of SPRY4-IT1, five pairs of primer were designed across the 1000 bp of promoter region. Chromatin immunoprecipitation assays indicated that EZH2 could directly bind to the *SPRY4-IT1* promoter region (−192 to −362 bp) and mediate H3K27me3 modification (Figures 2d and e), which was supported by the fact that knockdown of EZH2 expression reduced EZH2 binding and H3K27me3 modification (Figures 2f and g). These data indicated that EZH2-mediated H3K27me3 modification has a key role in the repression of *SPRY4-IT1* expression.

### *SPRY4-IT1* inhibits NSCLC cell proliferation and induces apoptosis

**Figure 3:**
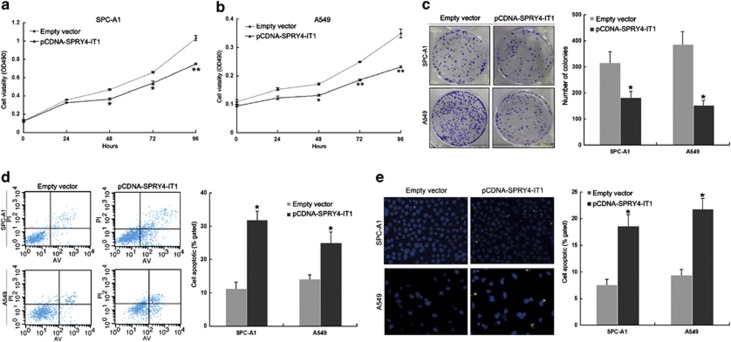
Effects of *SPRY4-IT1* on NSCLC cell proliferation and apoptosis *in vitro*. SPC-A1 and A549 cells were transfected with pCDNA-SPRY4-IT1. (**a** and **b**) MTT assays were used to determine the cell viability for pCDNA-SPRY4-IT1-transfected SPC-A1 and A549 cells. Values represent the mean±S.D. from three independent experiments. (**c**) Colony-forming assays were conducted to determine the colony-forming efficiency of pCDNA-SPRY4-IT1-transfected SPC-A1 and A549 cells. (**d**) Apoptosis was determined by flow cytometry. (**e**) Apoptosis was determined by Tunel staining, Blue, cell nucleus; Green, apoptotic cell. UL, necrotic cells; UR, terminal apoptotic cells; LR, early apoptotic cells. *P<0.05 and **P<0.01

To assess the biological role of *SPRY4-IT1* in NSCLC, we investigated the effects of *SPRY4-IT1* overexpression on the proliferation and apoptosis of SPC-A1 and A549 cells. Our qPCR results revealed that *SPRY4-IT1* expression was significantly upregulated compared with control cells (Supplementary Figure S2a), and MTT assays showed that the growth of SPC-A1, A549, and H1299 cells transfected with pCDNA-SPRY4-IT1 was impaired compared with control cells (Figures 3a and b and Supplementary Figure S3a). Colony formation assay results revealed that clonogenic survival was inhibited following overexpression of *SPRY4-IT1* in SPC-A1 and A549 cells (Figure 3c), while flow cytometry analysis and TUNEL staining showed that upregulation of *SPRY4-IT1* expression promoted apoptosis in these cells (Figures 3d and e).

### *SPRY4-IT1* inhibits NSCLC cells tumorigenesis *in vivo*

**Figure 4:**
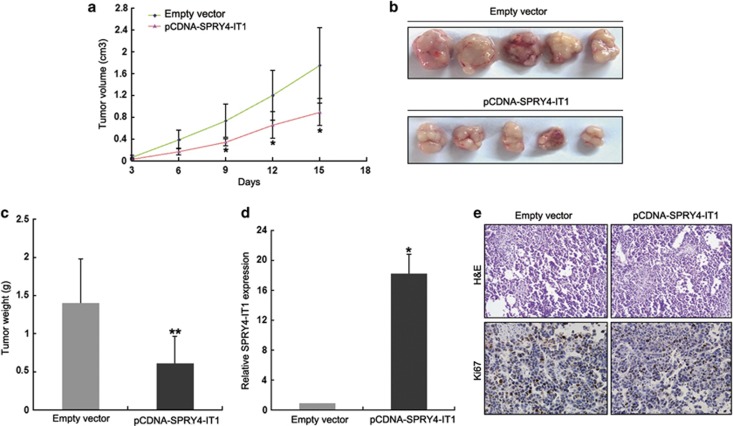
Overexpression of *SPRY4-IT1* suppresses tumor growth *in vivo*. (**a** and **b**) Tumor volume was calculated every 3 days after the injection of SPC-A1 cells stably transfected with pCDNA-SPRY4-IT1 or empty vector. Error bars indicate S.D. (**c**) Tumor weights are represented as means of tumor weights±S.D. (**d**) qPCR analysis of *SPRY4-IT1* expression in tumor tissues formed from SPC-A1/SPRY4-IT1 and SPC-A1/NC. (**e**) Tumors developed from pCDNA-SPRY4-IT1-transfected SPC-A1 cells showed lower Ki67 protein levels than tumors developed from control cells. Upper: hematoxylin and eosin (HE) staining; lower: immunostaining. *P<0.05, **P<0.01 and n.s., not significant

To explore whether the level of *SPRY4-IT1* expression could affect tumorigenesis, SPC-A1 cells stably transfected with pCDNA-SPRY4-IT1 or empty vector were inoculated into female nude mice. Eighteen days after the injection, the tumors formed in the pCDNA-SPRY4-IT1 group were substantially smaller than those in the control group (Figures 4a and b). Moreover, the mean tumor weight at the end of the experiment was markedly lower in the pCDNA-SPRY4-IT1 group (0.62±0.35 g) compared with the empty vector group (1.41±0.57 g) (Figure 4c). qPCR analysis found that the levels of *SPRY4-IT1* expression in tumor tissues formed from pCDNA-SPRY4-IT1 cells were higher than in tumors formed in the control group (Figure 4d). Tumors formed from pCDNA-SPRY4-IT1-transfected SPC-A1 cells exhibited decreased positivity for Ki67 than those from control cells (Figure 4e), while cells stably transfected with SPRY4-IT1 showed decreased proliferation *in vitro* (Supplementary Figure S3b). These findings indicate that overexpression of *SPRY4-IT1* inhibits tumor growth *in vivo*.

### *SPRY4-IT1* inhibits the migration and invasion of NSCLC cells

**Figure 5:**
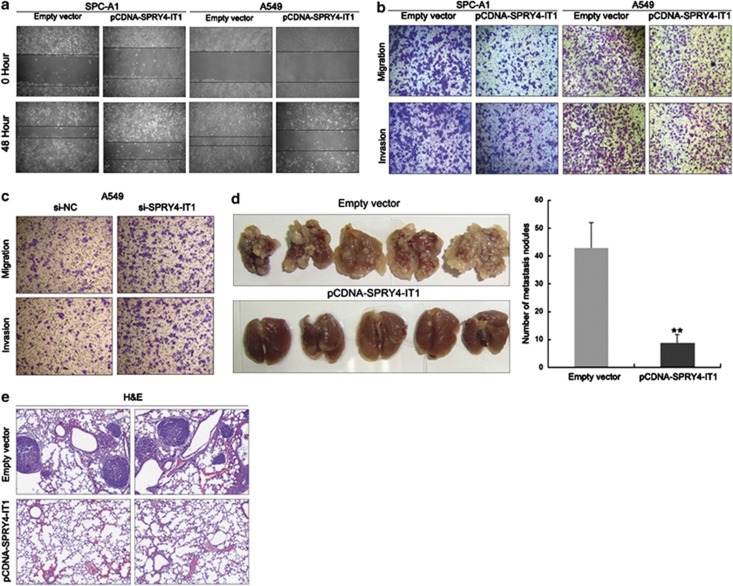
Effects of *SPRY4-IT1* on NSCLC cell migration, invasion, and metastasis. SPC-A1 and A549 cells were transfected with pCDNA-SPRY4-IT1. (**a**) Wound-healing assays were used to investigate the migratory ability of NSCLC cells. (**b**) Transwell assays were used to investigate the changes in migratory and invasive abilities of NSCLC cells. (**c**) Transwell assays were conducted to determine the migratory and invasive abilities of si-SPRY4-IT1-transfected A549 cells. (**d**) Analysis of an experimental metastatic animal model was performed by injecting A549 cells stably transfected with *SPRY4-IT1* into nude mice. Lungs from mice in each experimental group, with the numbers of tumor nodules on lung surfaces, are shown. (**e**) Visualization of the entire lung and hematoxylin and eosin (HE)-stained lung sections. *P<0.05 and **P<0.01

A wound-healing assay conducted on cells transfected with pCDNA-SPRY4-IT1 showed that they underwent a slower closing of scratch wounds compared with control cells (Figure 5a). We then used Matrigel (Sigma-Aldrich, St. Louis, MO, USA) to evaluate cancer cell invasion, and found that invasion of SPC-A1, A549, and H1299 cells was reduced by 52% following upregulation of *SPRY4-IT1* expression, while increased *SPRY4-IT1* expression levels impeded the migration of all three cell types by approximately 65% compared with controls, as shown by Transwell assays (Figure 5b and Supplementary Figure S3c).

### Knockdown of *SPRY4-IT1* expression promotes NSCLC cell invasion

To determine whether inhibition of *SPRY4-IT1* expression could promote NSCLC cell proliferation and invasion, we performed targeted knockdown of *SPRY4-IT1* expression using RNA interference in A549 cells (Supplementary Figure S2b). MTT assays revealed that downregulation of *SPRY4-IT1* expression did not affect cell proliferation (Supplementary Figure S2c); however, decreased *SPRY4-IT1* expression levels promoted A549 cell migration and invasion *in vitro* (Figure 5c).

### *SPRY4-IT1* suppresses NSCLC cell metastasis *in vivo*

To validate the effects of *SPRY4-IT1* on the metastasis of NSCLC cells *in vivo*, A549 cells stably transfected with pCDNA-SPRY4-IT1 were injected into nude mice. Metastatic nodules on the surface of the lungs were counted after 7 weeks. Ectopic overexpression of *SPRY4-IT1* reduced the number of metastatic nodules compared with the control group (Figure 5d). This difference was further confirmed following examination of the entire lungs, and through hematoxylin and eosin staining of lung sections (Figure 5e). Moreover, cells stably transfected with *SPRY4-IT1* showed decreased migration and invasion *in vitro* (Supplementary Figure S3d). Our *in vivo* data therefore complemented the results of functional *in vitro* studies involving *SPRY4-IT1*.

### Knockdown of EZH2 induces NSCLC cell growth arrest *in vitro* and *in vivo*

**Figure 6:**
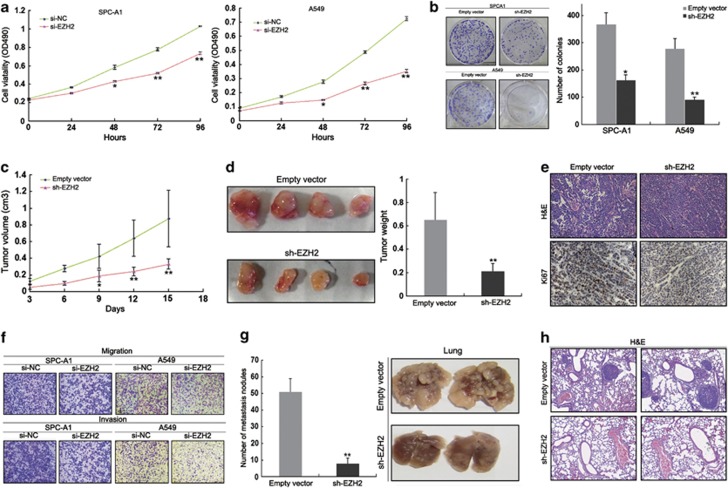
Knockdown of EZH2 expression inhibits NSCLC cell proliferation and metastasis *in vitro* and *in vivo*. (**a**) MTT assays were used to determine the cell viability of si-EZH2-transfected SPC-A1 and A549 cells. Values represent the mean±S.D. from three independent experiments. (**b**) Colony-forming assays were conducted to determine the colony-forming efficiency of sh-EZH2-transfected SPC-A1 and A549 cells. (**c**) Tumor volume was calculated every 3 days after the injection of SPC-A1 cells stably transfected with pCDNA-SPRY4-IT1 or empty vector. Error bars indicate S.D. (**d**) Tumor weights are represented as means of tumor weights±S.D. (**e**) Tumors developed from pCDNA-SPRY4-IT1-transfected SPC-A1 cells showed lower Ki67 protein levels than tumors developed from control cells. Upper: hematoxylin and eosin (HE) staining; lower: immunostaining. (**f**) Transwell assays were conducted to determine the migratory and invasive abilities of si-EZH2-transfected NSCLC cells. (**g**) Analysis of an experimental metastastic animal model was performed by injecting A549 cells stably transfected with EZH2 knockdown into nude mice. Lungs from mice in each experimental group, with the numbers of tumor nodules on lung surfaces, are shown. (**h**) Visualization of the entire lung and hematoxylin and eosin (HE)-stained lung sections. *P<0.05, **P<0.01

As an important regulatory factor of *SPRY4-IT1* expression, we next set out to validate whether EZH2 could also affect NSCLC cell proliferation. The MTT assay showed that inhibition of EZH2 expression significantly impaired NSCLC cell proliferation, while colony formation assay results revealed that clonogenic survival was inhibited following knockdown of EZH2 expression (Figures 6a and b). To further explore whether the inhibition of EZH2 expression could affect tumorigenesis, SPC-A1 cells stably transfected with sh-EZH2 or empty vector were inoculated into female nude mice. Eighteen days after the injection, tumors formed in the sh-EZH2 group of mice were substantially smaller than those in the control group (Figure 6c). Moreover, the mean tumor weight at the end of the experiment was significantly lower in the sh-EZH2 group (0.213±0.066 g) compared with the empty vector group (0.655±0.231 g) (Figure 6d). Immunostaining of resected tumor tissues found that tumors formed from sh-EZH2-transfected SPC-A1 cells exhibited decreased positivity for Ki67 than those from control cells (Figure 6e).

### Effect of EZH2 downregulation on NSCLC cell invasion and metastasis

Transwell assays revealed that downregulation of EZH2 expression significantly impaired NSCLC cell migration and invasion (Figure 6f). To validate the effects of EZH2 on the metastasis of NSCLC cells *in vivo*, A549 cells stably transfected with sh-EZH2 were injected into nude mice. Metastatic nodules on the surface of the lungs were counted after 7 weeks, and EZH2 inhibition was shown to reduce the number of metastatic nodules compared with those in the control group (Figure 6g). This difference was further confirmed following examination of the entire lungs, and through hematoxylin and eosin staining of lung sections (Figure 6h). Further, cells stably transfected with sh-EZH2 showed decreased proliferation, migration, and invasion *in vitro* (Supplementary Figures S3b and S3d). Thus, our *in vivo* data again complemented the results of functional *in vitro* studies involving EZH2.

### *SPRY4-IT1* influences the NSCLC cell EMT

**Figure 7:**
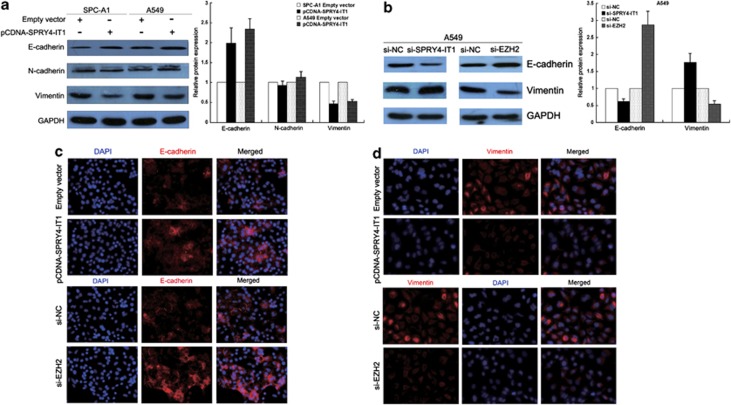
*SPRY4-IT1* overexpression suppresses NSCLC cell invasion and metastasis by affecting the EMT. (**a**) Western blot analysis of E-cadherin, N-cadherin, and vimentin expression in NSCLC cells treated with pCDNA-SPRY4-IT1. (**b**) Western blot analysis of E-cadherin and vimentin expression in A549 cells treated with si-SPRY4-IT1 or si-EZH2. (**c** and **d**) Immunofluorescence analysis of E-cadherin and vimentin expression in A549 cells treated with pCDNA-SPRY4-IT1 or si-EZH2. All experiments were performed in triplicate with three technical replicates. *P<0.05, **P<0.01

Neither the overexpression nor knockdown of SPRY4-IT1 had any significant change on SPRY4 expression (Supplementary Figure S2e). Previously, we found that the EMT was involved in lncRNA BANCR-mediated regulation of NSCLC cell invasion and metastasis. In the present study, we used western blotting to determine the expression of the EMT-induced markers E-cadherin, N-cadherin, and vimentin in cells overexpressing *SPRY4-IT1*. Increased *SPRY4-IT1* expression levels were found to induce E-cadherin expression and decrease that of vimentin, while decreased *SPRY4-IT1* expression inhibited E-cadherin expression and promoted vimentin expression (Figure 7a). Moreover, knockdown of EZH2 expression induced E-cadherin expression and decreased that of vimentin (Figure 7b). Immunofluorescence analysis revealed the same results in NSCLC cells (Figures 7c and d).

### Repression of *SPRY4-IT1* is potentially involved in the oncogenic function of EZH2

**Figure 8:**
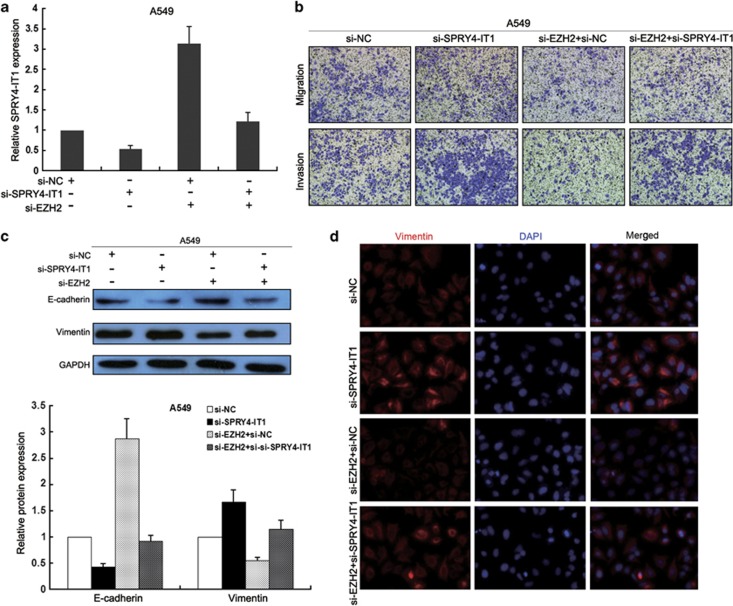
Inhibition of *SPRY4-IT1* partially rescues the impaired biological function of knockdown of EZH2 expression. A549 cells were cotransfected with si-EZH2 and si-SPRY4-IT1. (**a**) Analysis of *SPRY4-IT1* expression levels by qPCR following treatment of A549 cells with si-EZH2, si-SPRY4-IT1, or both. (**b**) Transwell assays were conducted to determine the migratory and invasive abilities of si-EZH2-, si-SPRY4-IT1-, or both si-EZH2- and si-SPRY4-IT1-transfected A549 cells. (**c**) Western blot analysis of E-cadherin and vimentin expression in A549 cells treated with si-EZH2, si-SPRY4-IT1, or both si-EZH2 and si-SPRY4-IT1. (**d**) Immunofluorescence analysis of E-cadherin and vimentin expression in A549 cells treated with si-EZH2, si-SPRY4-IT1, or both si-EZH2 and si-SPRY4-IT1. *P<0.05, **P<0.01

To investigate whether *SPRY4-IT1* is involved in the EZH2-induced increase in NSCLC cell proliferation and metastasis, we performed rescue experiments. A549 cells were cotransfected with si-EZH2 and si-SPRY4-IT1, and this was shown to rescue the increased expression of *SPRY4-IT1* induced by knockdown of EZH2 (Figure 8a). Moreover, a transwell assay indicated that the cotransfection could partially rescue si-EZH2-impaired migration and invasion in A549 cells (Figure 8b), while western blotting and immunofluorescence showed that it could also partially reverse the increase in E-cadherin and decrease in vimentin expression that occurred following the inhibition of EZH2 expression (Figures 8c and d). These data indicate that EZH2 promotes NSCLC cell proliferation and invasion through the downregulation of *SPRY4-IT1* expression.

## Discussion

Recently, many lncRNAs have been characterized and numerous pieces of evidence show that they play an important role in cancer pathogenesis, suggesting that they could provide new insights into the biology of this disease. However, NSCLC lncRNAs are still an emerging field, with only a handful of lncRNAs known to be involved in NSCLC tumorigenesis. One of these is metastasis-associated lung adenocarcinoma transcript 1 (*MALAT1*), also known as nuclear-enriched abundant transcript 2 (*NEAT2*), which is a highly conserved nuclear lncRNA and a predictive marker for metastasis development in lung cancer.^23^ We previously showed that lncRNA Hox transcript antisense intergenic RNA is upregulated in NSCLC, and that knockdown of its expression results in impaired cell invasion and metastasis through the regulation of *HOXA5* expression.^24^ In this study, we demonstrated that the expression of another lncRNA, *SPRY4-IT1*, is significantly downregulated in NSCLC tissues. Specifically, decreased *SPRY4-IT1* expression appears to be a significant, independent predictive value for NSCLC patients. Moreover, the upregulation of *SPRY4-IT1* expression led to the significant inhibition of cell proliferation, migration, invasion, and the promotion of apoptosis. Conversely, knockdown of *SPRY4-IT1* expression promoted cell migration and invasion. Increased *SPRY4-IT1* expression levels resulted in a significant reduction in the number of metastatic nodules on the lungs *in vivo*. These findings suggest that *SPRY4-IT1*has a direct role in the modulation of cell metastasis and NSCLC progression, and could be a useful novel prognostic or progression marker for NSCLC.

As more lncRNAs are studied, many have been shown to function as tumor suppressors involved in multiple cancers including the development of NSCLC. However, the regulatory factors that contribute to the repression of lncRNA have not been well documented. Typically, tumor suppressor genes are silenced by genetic or epigenetic alterations in cancer cells,^25^ but it is not clear whether epigenetic regulatory factors such as histone modification or DNA methylation also manipulate the expression of lncRNAs. Here, we found that the epigenetic factor EZH2 regulates lncRNA transcription. Knockdown of EZH2 expression significantly upregulated *SPRY4-IT1* expression, while chromatin immunoprecipitation assays showed that inhibition of EZH2 expression prevented its binding to the *SPRY4-IT1* promoter region and reduced H3K27me3 modification, which upregulated *SPRY4-IT1* expression. Furthermore, EZH2 and *SPRY4-IT1* expression were negatively correlated with each other. These results, together with those from a recent study^12^ highlight the role of epigenetics in regulating lncRNA transcription.

EZH2 is often expressed in NSCLC, and its expression is associated with the early pathogenesis of squamous cell carcinoma and correlates with a more aggressive tumor behavior of lung adenocarcinomas.^20^ In this study, we demonstrated that knockdown of EZH2 expression could induce NSCLC cell growth arrest and impair cell invasion and metastasis both *in vitro* and *in vivo*. Previous studies indicated that EZH2 is involved in regulating the proliferation and invasion of multiple cancer cells by suppressing tumor suppressor genes or the transcription of microRNAs. We also found that EZH2 represses lncRNAs, especially *SPRY4-IT1* transcription, as a means of contributing to NSCLC development. More importantly, knockdown of *SPRY4-IT1* could reverse the inhibition of the EZH2 expression-mediated impairment of NSCLC cell migration, invasion, and the EMT process. These data therefore confirm that *SPRY4-IT1* is a key regulatory factor underlying the EZH2 pathway.

To explore the molecular mechanism by which *SPRY4-IT1* contributes to the proliferation and metastasis of NSCLC, we investigated potential target proteins involved in cell motility and matrix invasion. We previously found that lncRNAs regulate NSCLC cell proliferation and metastasis by affecting the EMT process (data not shown). Hallmarks of EMT are the loss of E-cadherin expression and the aberrant expression of N-cadherin and vimentin.^26, 27, 28^ Therefore, we determined the protein levels of these EMT-induced markers following *SPRY4-IT1* overexpression or the inhibition of *SPRY4-IT1* or EZH2. Our results indicated that *SPRY4-IT1* mediated inhibitory effects on NSCLC cell migration, invasion, and metastasis suppression, possibly by affecting the EMT. As a central differentiation process, EMT allows for the remodeling of tissues during the early stages of embryogenesis, and is implicated in the promotion of tumor cell invasion and metastasis.^29^ It has also been proposed to be a mechanism for promoting the detachment of cancer cells from primary tumors, and to be associated with poor clinical outcome in NSCLC.^30, 31, 32^ Therefore, as regulators of EMT, lncRNAs could be suitable candidates for intervention in the treatment of cancer.

Although only a small number of functional lncRNAs have been well characterized to date, they have been shown to regulate gene expression at various levels, including chromatin modification, transcription, and post-transcriptional processing.^33, 34, 35^ Here, the possible mechanisms that underlie such regulatory behaviors still remain to be fully understood despite our observation of *SPRY4-IT1*-induced NSCLC cell growth arrest and regulation of the EMT phenotype. Further investigation into the *SPRY4-IT1* molecular and biological functions controlling EMT will undoubtedly be important in understanding the molecular biology of NSCLC metastasis and progression.

In conclusion, the expression of *SPRY4-IT1* was significantly decreased in NSCLC tissues, which was at least partially mediated by EZH2, suggesting that its downregulation may be a negative prognostic factor for NSCLC patients, indicative of poor survival rates, and a higher risk for cancer metastasis. We showed that *SPRY4-IT1* possibly regulates the invasive and metastatic ability of NSCLC cells, partially through its regulation of the EMT. Our findings further the understanding of NSCLC pathogenesis, and facilitate the development of lncRNA-directed diagnostics and therapeutics against cancers.

## Materials and Methods

### Tissue collection

We obtained 121 paired NSCLC and adjacent nontumor lung tissues from patients who underwent surgery at Jiangsu Province Hospital between 2008 and 2010, and who were diagnosed with NSCLC (stages I, II, and III) based on histopathological evaluation. Clinicopathological characteristics, including tumor node metastasis staging, were recorded. No local or systemic treatment was conducted in these patients before surgery. All collected tissue samples were immediately snap-frozen in liquid nitrogen and stored at −80 °C until required. Our study was approved by the Research Ethics Committee of Nanjing Medical University, China. Written informed consent was obtained from all patients.

### Cell lines

Five NSCLC adenocarcinoma cell lines (A549, SPC-A1, NCI-H1975, NCI-H1299, and NCI-H1650), an NSCLC squamous carcinomas cell line (SK-MES-1), and a normal human bronchial epithelial cell line (16HBE) were purchased from the Institute of Biochemistry and Cell Biology of the Chinese Academy of Sciences (Shanghai, China). A549, SK-MES-1, NCI-H1975, NCI-H1299, NCI-H1650, and 16HBE cells were cultured in RPMI 1640 medium, and SPC-A1 cells were cultured in DMEM (GIBCO-BRL, Invitrogen, Carlsbad, CA, USA) medium supplemented with 10% fetal bovine serum (FBS), 100 U/ml penicillin, and 100 mg/ml streptomycin (Invitrogen, Carlsbad, CA, USA) at 37 °C/5% CO_2_.

### RNA extraction and qPCR assays

Total RNA was isolated with TRIzol reagent (Invitrogen, Carlsbad, CA, USA) according to the manufacturer's instructions. Total RNA (500 ng) was reverse transcribed in a final volume of 10 *μ*l using random primers under standard conditions for the PrimeScript RT reagent Kit (TaKaRa, Dalian, China). We used the SYBR Premix Ex Taq ((TaKaRa, Dalian, China) to determine *SPRY4-IT1* expression levels, following the manufacturer's instructions. Results were normalized to the expression of glyceraldehyde-3-phosphate dehydrogenase (*GAPDH*). The specific primers used are presented in Supplementary Table 4. qPCR assays were conducted on an ABI 7500, and results were analyzed and expressed relative to threshold cycle (CT) values, then converted to fold changes.

### Plasmid generation

The *SPRY4-IT1* sequence was synthesized and subcloned into the pCDNA3.1 vector (Invitrogen, Shanghai, China). Ectopic expression of *SPRY4-IT1* was achieved through pCDNA-SPRY4-IT1 transfection, with an empty pCDNA vector used as a control. The expression levels of *SPRY4-IT1* were detected by qPCR.

### Cell transfection

Plasmid vectors (pCDNA3.1-SPRY4-IT1, and pCDNA3.1) for transfection were prepared using DNA Midiprep or Midiprep kits (Qiagen, Hilden, Germany), and transfected into SPC-A1 or A549 cells. The small interfering RNAs si-SPRY4-IT1, si-EZH2, or si-NC were transfected into SPC-A1 or A549 cells (Supplementary Table 4). A549 or SPC-A1 cells were grown in 6-well plates until confluent, then transfected with Lipofectamine 2000 (Invitrogen, Shanghai, China) according to the manufacturer's instructions. At 48 h post transfection, cells were harvested for qPCR or western blot analysis.

### Cell proliferation assays

Cell proliferation was monitored using a Cell Proliferation Reagent Kit I (MTT) (Roche Applied Science, Penzberg, Germany). A549 cells transfected with si-SPRY4-IT1 (3000 cells/well), and A549 or SPC-A1 cells transfected with pCDNA-SPRY4-IT1 were grown in 96-well plates. Cell proliferation was assessed every 24 h following the manufacturer's protocol. All experiments were performed in quadruplicate. For colony formation assays, pCDNA-SPRY4-IT1-transfected SPC-A1 or A549 cells (*n*=500) were placed in 6-well plates and maintained in media containing 10% FBS. The medium was replaced every 4 days; after 14 days, cells were fixed with methanol and stained with 0.1% crystal violet (Sigma-Aldrich). Visible colonies were then counted. For each treatment group, wells were assessed in triplicate.

### Flow cytometry analysis of apoptosis

SPC-A1 and A549 cells were harvested 48 h post transfection by trypsinization. After staining with FITC-Annexin V and propidium iodide, cells were analyzed by flow cytometry (FACScan; BD Biosciences) using CellQuest software (BD Biosciences, San Jose, CA, USA). Cells were discriminated into viable cells, dead cells, early apoptotic cells, and apoptotic cells. The ratio of early apoptotic cells was compared with that for controls from each experiment. All samples were assayed in triplicate.

### Wound-healing assay

A total of 3 × 10^5^ cells were seeded in 6-well plates, cultured overnight, and transfected with pCDNA-SPRY4-IT1 or the control vector. Once cultures reached 85% confluency, the cell layer was scratched with a sterile plastic tip and washed with culture medium, then cultured for 48 h with medium containing 1% FBS. At different time points, images of the plates were acquired using a microscope.

### Cell migration and invasion assays

For the migration assays, at 48 h post transfection, 5 × 10^4^ cells in serum-free media were placed into the upper chamber of an insert (8-*μ*m pore size; Millipore, Billerica, MA, USA). For the invasion assays, 1 × 10^5^ cells in serum-free medium were placed into the upper chamber of an insert coated with Matrigel. Medium containing 10% FBS was added to the lower chamber. After incubation for 24 h, the cells remaining on the upper membrane were removed with cotton wool. Cells that had migrated or invaded through the membrane were stained with methanol and 0.1% crystal violet, imaged, and counted using an IX71 inverted microscope (Olympus, Tokyo, Japan). Experiments were independently repeated three times.

### Tumor formation assay in a nude mouse model

Female athymic BALB/c nude mice (4 weeks old) were maintained under pathogen-free conditions and manipulated according to protocols approved by the Shanghai Medical Experimental Animal Care Commission. SPC-A1 cells were stably transfected with pCDNA-SPRY4-IT1 and empty vector, and harvested from 6-well cell culture plates, washed with phosphate-buffered saline, and resuspended at a concentration of 1 × 10^8^ cells/ml. A total of 100 *μ*l of suspended cells was subcutaneously injected into a single side of the posterior flank of each mouse. Tumor growth was examined every 3 days, and tumor volumes were calculated using the equation *V*=0.5 × *D* × *d*^2^ (V, volume; D, longitudinal diameter; d, latitudinal diameter). At 18 days post injection, mice were euthanized, and the subcutaneous growth of each tumor was examined. This study was carried out in strict accordance with the recommendations in the Guide for the Care and Use of Laboratory Animals of the National Institutes of Health. The protocol was approved by the Committee on the Ethics of Animal Experiments of the Nanjing medical University.

### Tail vein injections into athymic mice

Athymic male mice (4 weeks old) were purchased from the Animal Center of the Chinese Academy of Science (Shanghai, China) and maintained in laminar flow cabinets under specific pathogen-free conditions. A549 cells stably transfected with pCDNA-SPRY4-IT1 or the empty vector were harvested, washed with phosphate-buffered saline, and resuspended at 2 × 10^7^ cells/ml. Suspended cells (0.1 ml) were injected into the tail veins of nine mice, which were killed 7 weeks after injection. The lungs were removed and photographed, and visible tumors on the lung surface were counted. This study was carried out in strict accordance with the Guide for the Care and Use of Laboratory Animals of the National Institutes of Health. Our protocol was approved by the Committee on the Ethics of Animal Experiments of Nanjing Medical University. All surgery was performed under sodium pentobarbital anesthesia, and all efforts were made to minimize suffering.^36^

### Western blotting analysis

Cells were lysed using RIPA protein extraction reagent (Beyotime, Beijing, China) supplemented with a protease inhibitor cocktail (Roche, Pleasanton, CA, USA) and phenylmethylsulfonyl fluoride (Roche). The concentration of proteins was determined using the Bio-Rad (Hercules, CA, USA) protein assay kit. Protein extracts (50 *μ*g) were separated by 10% SDS-polyacrylamide gel electrophoresis, then transferred to nitrocellulose membranes (Sigma-Aldrich) and incubated with antibodies. Autoradiograms were quantified by densitometry using Quantity One software (Bio-Rad) using GAPDH as a control. Antibodies (1 : 1000 dilution) against E-cadherin and N-cadherin were purchased from BD Biosciences. Antibodies against vimentin was purchased from Cell Signaling Technology (Danvers, MA, USA).

### Fluorescence immunohistochemistry

Tissues were fixed in 4% paraformaldehyde for 24–36 h following a standard protocol, then dehydrated and embedded in paraffin. Sections (5 *μ*m) were mounted on glass slides (Fisher Scientific, Beijing, China). Mouse anti-E-cadherin and anti-N-cadherin polyclonal antibodies (1 : 100; BD Biosciences) were used as primary antibodies, with TRITC-labeled anti-Rabbit IgG (1 : 200; Sigma-Aldrich) used as a secondary antibody. Sections were mounted onto slides using Gel Mount Aqueous Mounting Medium (G0918, Sigma-Aldrich) and examined with an Olympus BX51 microscope (Olympus, Tokyo, Japan).

### Statistical analysis

Student's *t*-test (two tailed), one-way ANOVA, and the Mann–Whitney U test were used to analyze data with SPSS 16.0 software (IBM, Chicago, IL, USA). *P*-values of less than 0.05 were considered statistically significant.

## Abbreviations

EMT: epithelial–mesenchymal transition
*EZH2*: enhancer of zeste homolog 2
*HOTAIR*: Hox transcript antisense intergenic RNA
H3K27me3: lysine 27 residue of histone H3 trimethylation
lncRNAs: long noncoding RNAs
MALAT1: Metastasis-associated lung adenocarcinoma transcript 1
NSCLC: non-small-cell lung cancer
NEAT2: nuclear-enriched autosomal transcript 2
*SPRY4*: sprouty homolog 4
*SPRY4-IT1*: SPRY4 intronic transcript 1
