# Supplementary figures and images for: EZH2-mediated epigenetic suppression of long noncoding RNA SPRY4-IT1 promotes NSCLC cell proliferation and metastasis by affecting the epithelial–mesenchymal transition
Source: Cell Death Dis. 2014 Jun 26;5(6):e1298–. doi: 10.1038/cddis.2014.256 (PMC4611729; doi:10.1038/cddis.2014.256)

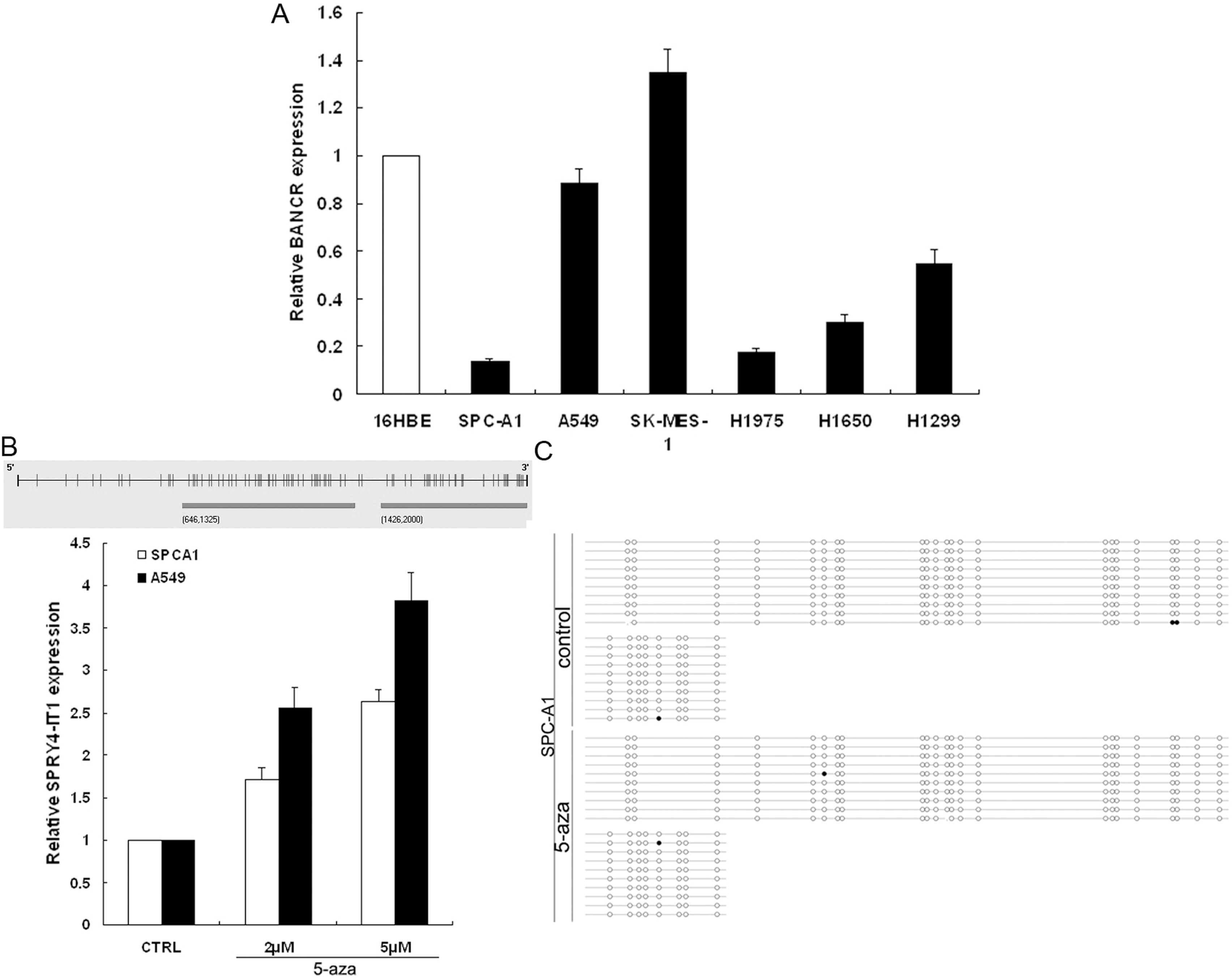

Supplement: Supplementary Figure S1 [file cddis2014256x1.tif]

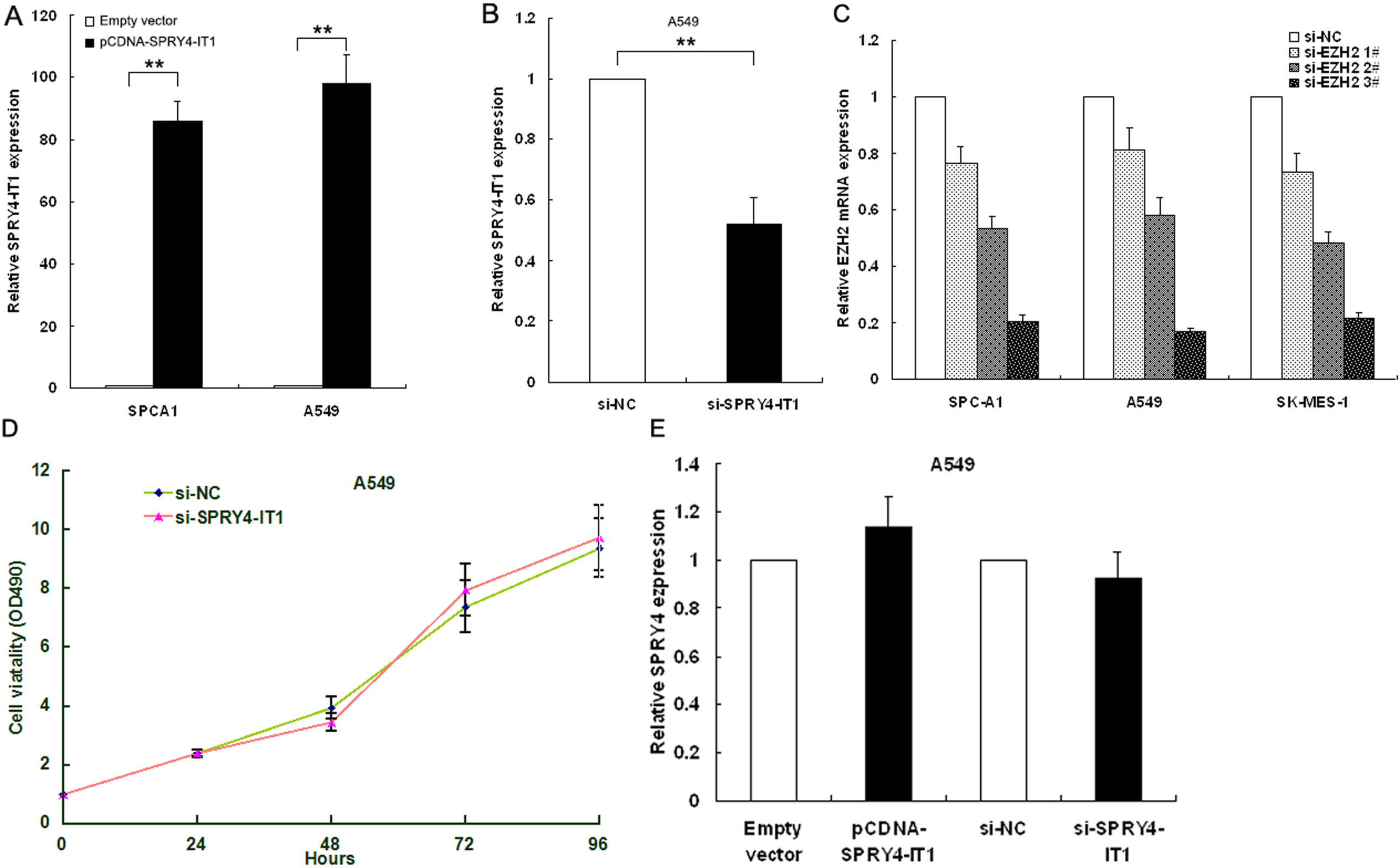

Supplement: Supplementary Figure S2 [file cddis2014256x2.tif]

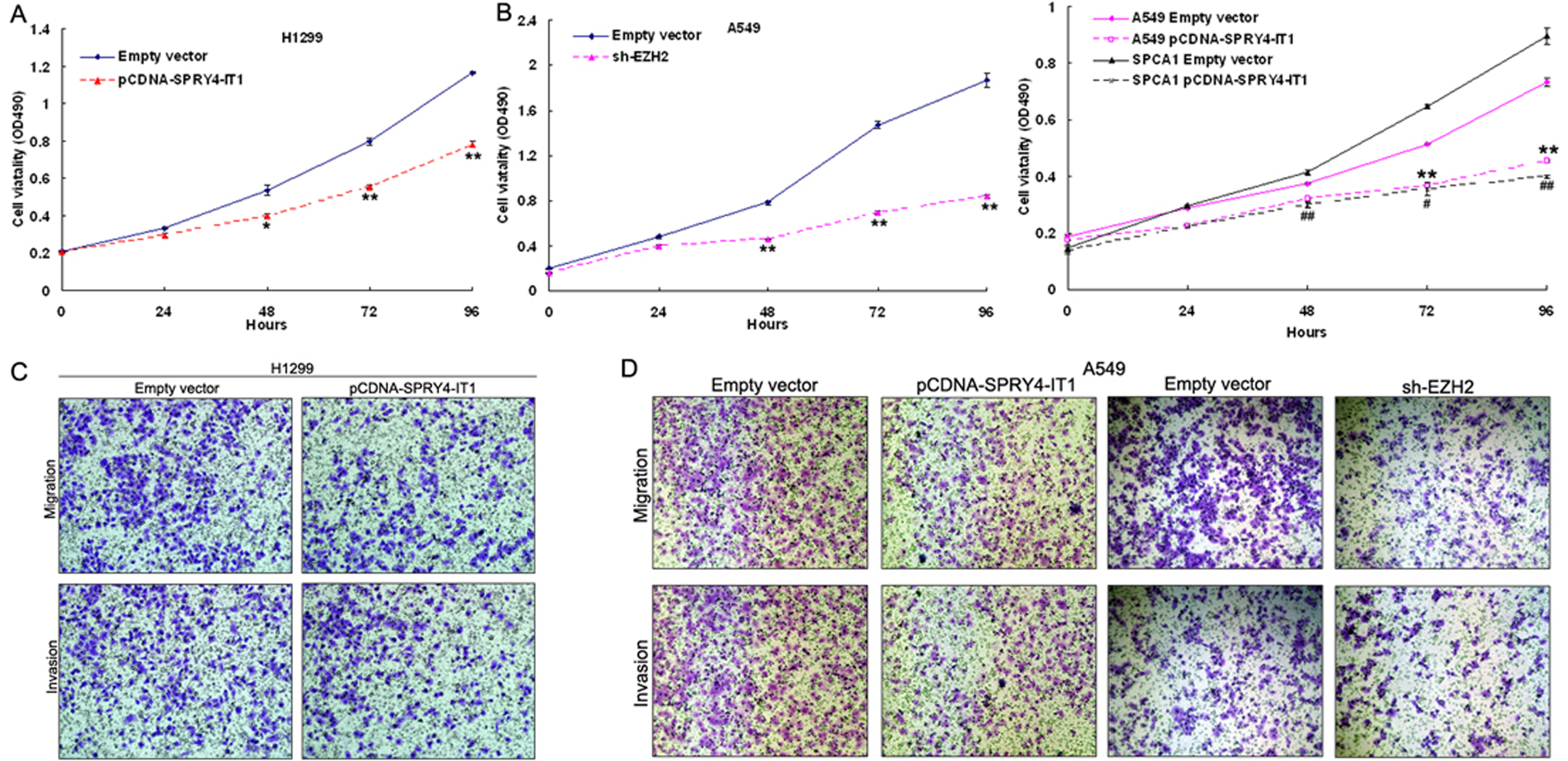

Supplement: Supplementary Figure S3 [file cddis2014256x3.tif]
