# Supplementary material for: EZH2-mediated epigenetic suppression of long noncoding RNA SPRY4-IT1 promotes NSCLC cell proliferation and metastasis by affecting the epithelial–mesenchymal transition
Source: Cell Death Dis. 2014 Jun 26;5(6):e1298–. doi: 10.1038/cddis.2014.256 (PMC4611729; doi:10.1038/cddis.2014.256)
Supplement: Supplementary Figure Legends [file cddis2014256x4.doc]

**Supplementary Titles and legends to figures**

**Supplementary Figure 1** Effects of DNA methylation on *SPRY4-IT1* expression. (**a**) qPCR analysis of *SPRY4-IT1* expression levels in NSCLC cell lines (A549, SPC-A1, H1650, H1299, H1975, and SK-MES-1) compared with the normal bronchial epithelial cell line 16HBE. (**b**) The level of *SPRY4-IT1* expression in SPC-A1 and A549 cells following 5-Aza (0, 5, 10 µM) treatment. (**c**) The methylation status of the *SPRY4-IT1* CpG island was assessed by bisulfite sequencing in SPCA1 cells. Open and filled squares denote unmethylated and methylated CpG sites, respectively. Each row represents a single clone. **P*<0.05; ***P*<0.01

**Supplementary Figure 2** Exogenous modulation of *SPRY4-IT1* in NSCLC cells. (**a**) qPCR analysis of *SPRY4-IT1* expression levels in SPC-A1 and A549 cells transfected with pCDNA-SPRY4-IT1 or empty vector. (**b**) qPCR analysis of *SPRY4-IT1* expression levels in A549 cells transfected with si-SPRY4-IT1 or si-NC. (**c**) qPCR analysis of EZH2 expression levels in SPC-A1, A549, and SK-MES-1 cells transfected with si-EZH2 or si-NC. (**d**) MTT assays were used to determine the cell viability for si- SPRY4-IT1-transfected SPC-A1 cells. (**e**) qPCR analysis of *SPRY4-IT1* expression levels in A549 cells transfected with pCDNA-SPRY4-IT1 or si-SPRY4-IT1. ***P*<0.01

**Supplementary Figure 3** Effects of EZH2 and SPRY4-IT1 on NSCLC cell proliferation, migration and invasion. (**a**) MTT assays were used to determine the cell viability for pCDNA-SPRY4-IT1-transfected H1299 cells. Values represent the mean ± s.d. from three independent experiments. (**b**) MTT assays were used to determine the cell viability for NSCLC cells stably transfected with sh-EZH2 or pCDNA-SPRY4-IT1. Values represent the mean ± s.d. from three independent experiments. (**c**) Transwell( assays were conducted to determine the migratory and invasive abilities of pCDNA-SPRY4-IT1-transfected H1299 cells. (**d**) Transwell( assays were conducted to determine the migratory and invasive abilities of cells stably transfected with pCDNA-SPRY4-IT1 or sh-EZH2 NSCLC.**P*<0.05; ***P*<0.01
