# Supplementary material for: EZH2-mediated epigenetic suppression of long noncoding RNA SPRY4-IT1 promotes NSCLC cell proliferation and metastasis by affecting the epithelial–mesenchymal transition
Source: Cell Death Dis. 2014 Jun 26;5(6):e1298–. doi: 10.1038/cddis.2014.256 (PMC4611729; doi:10.1038/cddis.2014.256)
Supplement: Supplementary Table 1 [file cddis2014256x5.doc]

**Table 1** Correlation between SPRY4-IT expression and clinicopathological characteristics of NSCLC patients。

| **Characteristics** | **SPRY4-IT** | | **P** |
| --- | --- | --- | --- |
|  | High No. cases (60) | Low No. cases (61) | Chi-squared test P-value |
| **Age(years)** |  |  | 0.522 |
| ≤65 | 33 | 30 |  |
| >65 | 27 | 31 |  |
| **Gender** |  |  | 0.298 |
| Male | 39 | 34 |  |
| Female | 21 | 27 |  |
| **Histological subtype** |  |  | 0.317 |
| Squamous cell carcinoma | 33 | 39 |  |
| Adenocarcinoma | 27 | 22 |  |
| **TNM Stage** |  |  | <0.001* |
| Ia + Ib | 28 | 9 |  |
| IIa + IIb | 19 | 21 |  |
| IIIa | 13 | 31 |  |
| **Tumor size** |  |  | 0.001* |
| ≤5cm | 39 | 21 |  |
| >5cm | 21 | 40 |  |
| **Lymph node metastasis** |  |  | 0.003* |
| Negative | 37 | 21 |  |
| Positive | 23 | 40 |  |
| **Smoking History** |  |  | 0.138 |
| Smokers | 44 | 37 |  |
| Never Smokers | 16 | 24 |  |

* Overall P<0.05
