# Supplementary material for: EZH2-mediated epigenetic suppression of long noncoding RNA SPRY4-IT1 promotes NSCLC cell proliferation and metastasis by affecting the epithelial–mesenchymal transition
Source: Cell Death Dis. 2014 Jun 26;5(6):e1298–. doi: 10.1038/cddis.2014.256 (PMC4611729; doi:10.1038/cddis.2014.256)
Supplement: Supplementary Table 2 [file cddis2014256x6.doc]

**Table 2** Univariate and multivariate analysis of progression-free survival in NSCLC patients (n=121)

| **Variables** | **Univariate analysis** | | | **Multivariate analysis** | | |
| --- | --- | --- | --- | --- | --- | --- |
|  | HR | 95% CI | p value | HR | 95% CI | p value |
| age | 1.056 | 0.672-1.657 | 0.814 |  |  |  |
| gender | 1.150 | 0.732-1.807 | 0.545 |  |  |  |
| smoker | 1.040 | 0.826-1.310 | 0.738 |  |  |  |
| Histological subtype | 0.976 | 0.779-1.223 | 0.833 |  |  |  |
| Chemotherapy | 0.888 | 0.707-1.114 | 0.303 |  |  |  |
| tumor size | 1.382 | 1.101-1.737 | 0.005* | 1.314 | 1.035-1.667 | 0.025* |
| lymph node metastasis | 0.680 | 0.540-0.856 | 0.001* | 0.741 | 0.583-0.943 | 0.015* |
| TNM stage (I vs. II or IIIa) | 1.418 | 1.093-1.839 | 0. 009* | 1.248 | 0.948-1.643 | 0.115 |
| SPRY4-IT expression | 0.319 | 0.198-0.512 | <0. 001* | 0.436 | 0.260-0.731 | 0.002* |

HR, hazard ratio; 95 % CI, 95 % conﬁdence interval

* Overall P<0.05
