# Supplementary material for: EZH2-mediated epigenetic suppression of long noncoding RNA SPRY4-IT1 promotes NSCLC cell proliferation and metastasis by affecting the epithelial–mesenchymal transition
Source: Cell Death Dis. 2014 Jun 26;5(6):e1298–. doi: 10.1038/cddis.2014.256 (PMC4611729; doi:10.1038/cddis.2014.256)
Supplement: Supplementary Table 3 [file cddis2014256x7.doc]

**Table 3** Univariate and multivariate analysis of overall survival in NSCLC patients (n=121)

| **Variables** | **Univariate analysis** | | | **Multivariate analysis** | | |
| --- | --- | --- | --- | --- | --- | --- |
|  | HR | 95% CI | p value | HR | 95% CI | p value |
| age | 1.238 | 0.721-2.126 | 0.439 |  |  |  |
| gender | 1.061 | 0.613-1.834 | 0.833 |  |  |  |
| smoker | 1.161 | 0.883-1.524 | 0.282 |  |  |  |
| Histological subtype | 0.931 | 0.709-1.223 | 0.607 |  |  |  |
| Chemotherapy | 0.855 | 0.649-1.127 | 0.268 |  |  |  |
| tumor size | 1.297 | 0.986-1.705 | 0.063 |  |  |  |
| lymph node metastasis | 0.678 | 0.513-0.897 | 0.006* | 0.770 | 0.577-1.028 | 0.076 |
| TNM stage (I vs. II or IIIa) | 1.674 | 1.185-2.364 | 0. 003* | 1.454 | 1.015-2.082 | 0.041* |
| SPRY4-IT expression | 0.345 | 0.195-0.611 | <0. 001* | 0.449 | 0.246-0.819 | 0.009* |

HR, hazard ratio; 95 % CI, 95 % conﬁdence interval

* Overall P<0.05
