# Supplementary material for: EZH2-mediated epigenetic suppression of long noncoding RNA SPRY4-IT1 promotes NSCLC cell proliferation and metastasis by affecting the epithelial–mesenchymal transition
Source: Cell Death Dis. 2014 Jun 26;5(6):e1298–. doi: 10.1038/cddis.2014.256 (PMC4611729; doi:10.1038/cddis.2014.256)
Supplement: Supplementary Table 5 [file cddis2014256x9.doc]

Supplementary table 5 Correlation between EZH2 expression and clinicopathological characteristics of NSCLC patients

| Characteristics | EZH2 |  | P |
| --- | --- | --- | --- |
|  | High No. cases (%) | Low No. cases (%) | Chi-squared test P-value |
| Age(years) |  |  | 1.0 |
| ≤65 | 11(54.7) | 11(50.0) |  |
| >65 | 12(45.3) | 12(50.0) |  |
| Gender |  |  | 0.475 |
| Male | 17(66.0) | 19(55.0) |  |
| Female | 6(34.0) | 4(45.0) |  |
| Histological subtype |  |  | 0.753 |
| Squamous cell carcinoma | 7(56.6) | 8(63.3) |  |
| Adenocarcinoma | 16(43.4) | 15(36.7) |  |
| TNM Stage |  |  | 0.042* |
| Ia + Ib | 3(47.2) | 10(15.0) |  |
| IIa + IIb | 7(32.1) | 7(35.0) |  |
| IIIa | 13(20.7) | 6(50.0) |  |
| Tumor size |  |  | 0.016* |
| ≤5cm | 5(66.0) | 13(35.0) |  |
| >5cm | 18(34.0) | 10(65.0) |  |
| Lymph node metastasis |  |  | 0.038* |
| Negative | 7(64.2) | 14(33.3) |  |
| Positive | 16(35.8) | 9(66.7) |  |
| Smoking History |  |  | 0.153 |
| Smokers | 16(64.2) | 20(60.0) |  |
| Never Smokers | 7(35.8) | 3(40.0) |  |

* Overall P<0.05
